# Chicago Medical Society

**Published:** 1886-11

**Authors:** 


### CHICAGO MEDICAL SOCIETY.

Officially Reported for THE ATLANTA MEDICAL AND SURGICAL JOURNAL.

STATED MEETING, Sept. 20, 1886.

Second Vice-President J. F. Todd, M. D., in the chair.

Professor W. E. Casselberry read a paper entitled

### PHARYNGEAL AND NASAL SURGERY BY THE GALVANO-CAUTERY, WITH REPORT OF CASES AND EXHIBITION OF APPARATUS.

New methods of utilizing the galvano-cautery are constantly being devised and recent improvements in batteries and electrodes, and the introduction of the local anæsthetic, cocaine, have materially extended its field of usefulness. In nasal, pharyngeal and even in laryngeal surgery its utility is particularly manifest. Pathological conditions of the upper respiratory passages, which have long baffled the most skillful therapeutics, are now effectually remedied through the medium of this agent.

CASE I.—Folliculous pharyngitis with hypertrophy in the angles of the pharynx. Dr. —— has suffered from fullness, soreness and pain in the pharyngeal region, culminating frequently in acute pharyngitis with extension of the inflammation to the middle ear. Examination disclosed two enormous hypertrophied follicular masses, occupying the latero-posterior angles of the pharnyx, each about three-fourths in length, of the thickness of a lead-pencil, and jutting inward and forward from behind the corresponding posterior pillar. In the normal state a whole chain of muco-lymphoid follicles ranges along in this location, and it is the hypertrophy of this entire row of glands on each side which is the chief feature of the case.

*Treatment.*—For the purpose of destroying these growths by the galvano-cautery an electrode was extemporized by uncoiling the wire end of a Fleming moxa-electrode, thus converting the coil into an elongated loop. This was so curved as to accurately approximate the mass. The loop heated instantly to a white heat by the action of the battery, produced by its slough, with but little pain and no hæmorrhage. After several operations, performed at proper intervals, the growth was entirely removed.

CASE 2.—Folliculous naso-pharyngitis or adenoid vegetations at the vault of the pharynx. Operations by the galvano-cautery écraseur. The symptoms were those of post-nasal catarrh. Examination, in addition to morbid conditions of other parts, revealed numerous hypertrophied follicular masses—so-called adenoid vegetations covering and filling up the vault up the pharynx; some of these appeared in the rhiniscopic mirror as pendant pear-shaped bodies; others were sessile, and still others had the appearance of several cockscombs crowded together.

*Treatment.*—Removal of the hypertrophied masses is the only satisfactory means of relief. This he first sought to accomplish with a Löwenberg cutting forceps, but the effort resulted unsatisfactorily. The cold snare and other methods were tried and abandoned, until finally he found that the object could be attained most easily and with the least pain and inconvenience to the patient by means of a galvano-cautery écraseur. He constructed an écraseur for the case by bending a fine pair of straight tubes, threaded with a platinum loop, to the proper curve to pass behind the velum palati and touch the hypertrophied mass. The naso-pharynx was sprayed with cocaine solution. Guided by the rhinoscopic image he introduced the écraseur and caused it to encircle one of the growths, then connecting the battery, the loop heated instantly to redness, was rapidly wound in on the windlass, and the vegetation thus severed at its base. There was no pain, no hæmorrhage, and but little subsequent irritation. The operations were repeated weekly until the growths were entirely removed.

CASE 3.—Malformation of the anterior pillars of the fauces. The symptoms were a sense of constriction in the pharynx and snoring. The malformation discovered was of the anterior pillars —*i. e.*, of the palato-glossal folds. These originated properly in the velum, but increasing in breadth they covered a considerable portion of the inner surface of the tonsils, and then a part only of the fold continuing in the normal pathway forward to the side of the tongue, the larger portion swept *backwards* and incorporated itself with the posterior pillar, forming a thick band, which continued downward in the latero-posterior angle of the inferiormost portion of the pharynx.

*Treatment.*—The overgrowth of each tonsil, together with the portion of the anterior pillar which covered it, was excised by means of the galvano-cautery écraseur, and at the same time the abnormal attachments of the anterior pillars to the pharyngeal wall were severed.

CASE 4.—Membranous occlusion of the posterior nares. Mr. E. suffered from complete obstruction of the left nasal chamber most violent headaches, vertigo, left-sided deafness and fetor of the breath. A tense membrane was found to cover the left choana almost completely, and another to partially occlude the right choana. These membranes were incised by the galvano-cautery knife electrode properly curved and introduced from behind. Complete relief to all symptoms. Certainly the galvano-cautery can be abused, and a word of caution is not unnecessary in this respect. Accurate diagnosis, precise indications for its use and a certain amount of skill on the part of the operator, are essentials to its judicious employment. The Fleming battery has advantages over any other that he has seen, not the least of which is the arrangement by which the plates may be immersed and withdrawn from the fluid by means of a pedal easily manipulated by one foot.

Dr. F. O. Stockton, in opening the discussion, said: I have listened with a great deal of pleasure this evening to the paper; the cases are all very interesting. It does not seem necessary to spoil an electrode in order to make one for a special case; it seems to me that the ordinary flat electrode, by simply bending it down, would answer the same purpose. The trouble is we must have new instruments for every case that comes up. Unfortunately, all practicing physicians, many of them who understand the throat and nose, are not so situated that they can run into an instrument maker's establishment at any time and have an electrode made, and I think it is wise to keep down to the standard instruments which can be bought at any time and which are always at hand. Another point I would like to speak of is the idea that follicular pharyngitis and adenoid vegetations are one and the same thing. Some authors consider them the same and others do not. Bosworth, of New York, claims that follicular pharyngitis and adenoid vegetations are one and the same. The battery which Dr. Casselberry shows is the one I have used in my practice for three years. I have tried a number of others and found this one the most satisfactory. A short time ago I saw a modification of it which I think is a little better ; it was devised by Dr. Sajous, of Philadelphia, but it is not on the market at present. Many of our prominent writers have rather discouraged people from using the galvano-cautery, as they say it requires a great deal of skill and knowledge, not only of the anatomy of the part we are working upon, but also of electricity and the management of instruments. Dr. Morell Mackenzie, of London, makes that objection to the galvano-cautery; in fact, he says that all, or nearly all, the operations can be performed by simpler means. Dr. Cohen, in his last work, also refers to the difficulty of managing it, but I think if they had used the instruments of to-day they would not have found that difficulty.

Dr. H. T. Gradle said : The doctor's paper was so complete there is nothing left for comment, except to deviate from the subject and take excursions into neighboring parts. It has been mentioned that galvano-cautery is not absolutely necessary. That may be true as far as the pharynx is concerned, but in the nose it is not. It would be almost impossible to do the same work as steadily by any other instrument on account of the excessive hæmorrhage that would occur ; these cases are unmanageable except with the cautery, and in most instances we can obtain relief both of local symptoms and obstruction of the nose, as well as the nervous symptoms maintained by the excessive turgidity. The question was asked whether hypertrophy of the pharyngeal tonsil could be identified with follicular pharyngits ; I should say not. We may have one or the other, but the follicles in the disease called follicular pharyngitis are much below the level of the gland. I have often found the gland hypertrophied into a very solid mass of about the consistency of the tonsils. The hypertrophy may of course have any extent ; it may be so slight as to leave room for doubt of its existence, while in other cases it is impossible to gain any view of the posterior choanal. In such cases nothing can be thought of but operation. I have used the cautery in the form of a white hot knife or hot snare for hypertrophy of the pharyngeal tonsil, but not satisfactorily. Where we have a distinctly pedunculated tumor it will do well enough to use the hot snare, but where the tonsil is hypertrophied into a solid mass, not at all movable with the probe, I have found very little success with the battery, except that its removal is not so painful as by other means. I have at last settled down on the method suggested by Trautman, viz., a sharp curette, as sharp as the instrument-maker can make it, the shank of which is bent at an angle of 140°. The operation is a barbarous one as far as appearances go, because it requires force. There is pretty free hæmorrhage, but the pain is not very great. Unless the person is pretty tolerant it is not easy to remove the entire tonsil in one sitting, but in children I have not had any greater difficulty in coaxing them to the second sitting than I had at first. The results are extremely satisfactory, especially in cases of ear disease entirely due to this trouble. I have had considerable experience with different forms of battery ; the kind which I should prefer and recommend is an accumulator, if a wire can be had from some electric light plant. I can speak well of the Mackintosh battery, but I prefer one in which the carbon plates are made of the electric light sticks. They are much harder than the ordinary carbon plates, and we can easily get a very large surface in the battery.

Dr. R. Tilley said: I have had some experience with the cautery and with batteries. I am now using the form of battery Dr. Gradle has referred to; I have the two cells attached so that I use them together, and have them so arranged that I can lift them up with a crank; they are thus very easily manipulated. I

have used this battery for about eighteen months, and it has always given me satisfaction. I would like to speak of a slight modification in the fluid which I have recently used; it consists in the use of bichromate of soda instead of bichromate of potash; the advantage is that when the fluid has been used for a considerable length of time, in using the potash there is always formed a large number of crystals in the bottom of the cell, which are very hard and require quite a little time to remove, and which are so closely connected with the cell that there is more or less danger of breaking the glass. In using soda instead of potash this does not occur; the compound thus formed is so soluble that no accumulation is formed at the bottom of the cell. I appreciate Dr. Stockton's remarks in regard to the increase in the number of instruments; I make all my own electrodes and use, practically, wire alone. I see no advantage in the knife over certain modifications of the wire. In operations in the post-nasal and pharyngeal regions, I almost always use the cautery to burn the tissues completely to the bone. The object that I have in view is to make a cicatricial tissue extending from the surface of the mucous membrane to the periosteum, so as to prevent that tumefaction which frequently occurs and fills up the nares. This tumefaction is quite remarkable sometimes and changes so readily that I have seen the anterior part of the turbinated bone swollen up, and a moment afterwards the swelling gone, so that there could not be any possible reason or justification for using the cautery from the appearance of the tissues at the moment. Yet when the patient lies down at night he finds the nose on the side on which he is lying so stuffed up that it is almost impossible to breathe. I have found that when the anterior part of the turbinated bone is burned well down to the bone that tumefaction rarely occurs afterwards. Then there is a necessity of looking after these burns, especially in the posterior regions. In the anterior aspect of the nose there is only a small amount of mucus coming over the part that is burned, and no difficulties follow cauterization; but when the burned area is situated far back the mucus flows over it and it not infrequently happens that unless the part is well attended to a certain amount of fungus growth develops in this region. I consider the aftertreatment an important factor in cauterizations. I would like to say a word in regard to these adenoid growths in the posterior nares that Dr. Gradle has referred to. Dr. Trautman, of Berlin, removes all these vegetations with the sharp spoon, and claims remarkable success. I have not seen anything like relatively the same number of cases in my practice that it would seem are met in practice in Berlin. I think adenoid growths are more rare in Chicago. With reference to their removal with the galvano écraseur, I should certainly be disposed to think that the ordinary wire écraseur would be more efficient than the sharp curette, and I think it could be more easily manipulated and more of the growth taken off at the same sitting than with the galvano-cautery.

Dr. W. E. Casselberry, in closing the discussion, said : There are some points on which I would like to add a few words of explanation. In reference to spoiling an electrode in order to make a single loop to treat a special case, I spoiled the moxa-electrode because it was an easier way to get the loop than to send to Philadelphia and have one made. As to the merits of the loop over a knife electrode, I think that in cauterizing the soft tissue, by taking a small elongated loop and pressing it into the part you get a slough produced by each parallel wire, and it produces a larger slough with less pain than can be obtained by the knife electrode ; the loop will sink further into the soft growth. If you use a plain knife electrode the slough produced at once under the flat surface of the electrode prevents further effect from its caustic action. I have not only employed the small loop in this particular case, but have found it useful in cases of hypertrophy of the inferior turbinated body where it was unnecessary to cut off a large section by means of the knife or écraseur. By simply taking the elongated loop and pressing it against the body, a considerable slough is produced in the centre ; after this has separated there remains an ulcer, and when the ulcer heals there is a cicatricial tissue, which afterwards binds the body down. In regard to the multiplicity of instruments, of course the multiplying of useless instruments is to be deprecated, but I would call to mind the fact that a large portion of the advance of medical science during the last half century has been directly due to the invention of new and improved instruments and apparatus. If those who devised the microscope, the ophthalmoscope, the laryngoscope, etc., had been satisfied with the "standard instruments" already in the market, we would not now have those most important forms of apparatus. A new instrument should always receive attention, and if it is a real improvement the inventor should be given due credit. The glandular structures of the vault of the pharynx, out of which adenoid vegetations are formed, and the follicles of the oro-pharynx may not be histologically identical, but they are very similar in structure ; they are both lymphoid tissues and both exhibit the same tendency to undergo the hypertrophic process and to form similar large masses which, if allowed to remain, produce morbid symptoms. I don't know that it is necessary to say anything more in regard to batteries ; there are a great many in the market. I am favorably impressed with the one I use ; it has never failed to work. Although it may not be detrimental to have the plates remain in the fluid, I have an uncomfortable feeling when I know that my plates are being spoiled and the fluid being used up faster than they need be.

The Sajous battery spoken of is an improvement on the Fleming in this respect the pedal is not so high from the floor. When one is operating, if he raises his foot to the height of eight inches he is liable to throw himself out of balance and lose his line of vision. The pedal of the Sajous battery is only about two or three inches from the floor and it is unnecessary to raise the heel off the floor to work it, thus maintaining the balance of the body. The Sajous plates also are corrugated, thus giving the most surface in the least bulk. But unfortunately the Sajous battery is not in the market. The one which Dr. Sajous uses he had made to order, but I have no doubt that it will be put upon the market soon. The various methods of operating for adenoid vegetations have all the same end in view, that is, the removal of the vegetations. The sharp curette is a good method if the patient will stand the blood, increase of pain and the length of the operation. In the case of children it is, I think, a rather difficult method, and requires considerable skill and fortitude on the part of the patient. The case I have related in this connection was a girl about fourteen years old. I first tried the forceps, but the pain in cutting off the adenoid vegetations was objected to very strenuously by the patient, and I was obliged to desist. Before I tried the hot snare I tried the cold snare, but I found that it took some time to draw up the steel wire and thus squeeze off the vegetation. After the adoption of the hot snare I had no difficulty. The mere introduction of the instrument caused no pain, and after getting it over the vegetation, in two seconds the growth was off, with but little pain and no hæmorrhage to frighten the patient. The treatment of hay-fever by the cautery method, I would like to discuss, but the lateness of the hour will not permit it.

---

PASTEURISM.—At a lecture given in the Sorbonne, Paris, on Sunday, October 10th, by Dr. Chautemps, of Paris, Vice-President of the Paris Municipal Council, on M. Pasteur's method, the lecturer stated that out of a total of 1,583 French patients, only 10 deaths occurred, of which 2 were attributable to accidental causes. He desired to deal only with the cases under treatment previous to August 1st in order to leave an ample margin of seventy days. They amounted to 1,276. Ten per cent. of these were bitten by dogs manifestly mad, and in 70 per cent. of the cases the dogs were scientifically shown to have been rabid. The remaining 20 per cent. were doubtful cases. Hence, out of 1,276 persons, 973 were bitten by dogs ascertained to have been mad. In the natural order of things, 155 deaths would have taken place, or 145 over the insignificant rate. Dr. Chautemps was satisfied M. Pasteur had saved 145 lives. Cases of rabies were of more frequent occurrence than was generally supposed. In the space of five months 81 cases of dog-bites came to the knowledge of the Paris Prefecture of Police. It was a significant fact that only 10 of M. Pasteur's patients died, while he (M. Pasteur) had heard, almost by accident, of 12 unvaccinated cases ending fatally. Several of his failures might be set down to the long interval that elapsed between the bite and treatment, but even this risk would no longer exist when Pasteurian virus stations were established in Russia, at New York, at Buenos Ayres and elsewhere.—*British Medical Journal, Oct. 16, 1886.*

